# Metformin promotes *in vitro* maturation of oocytes from aged mice by attenuating mitochondrial oxidative stress *via* SIRT3-dependent SOD2ac

**DOI:** 10.3389/fcell.2022.1028510

**Published:** 2022-10-25

**Authors:** Yongzhi Cao, Zhao Wang, Changming Zhang, Yuehong Bian, Xin Zhang, Xin Liu, Wendi Chen, Yueran Zhao

**Affiliations:** ^1^ Center for Reproductive Medicine, Shandong University, Jinan, Shandong, China; ^2^ Key Laboratory of Reproductive Endocrinology of Ministry of Education, Shandong University, Jinan, Shandong, China; ^3^ Shandong Key Laboratory of Reproductive Medicine, Jinan, Shandong, China; ^4^ Shandong Provincial Clinical Research Center for Reproductive Health, Jinan, Shandong, China; ^5^ Shandong Technology Innovation Center for Reproductive Health, Jinan, Shandong, China; ^6^ National Research Center for Assisted Reproductive Technology and Reproductive Genetics, Shandong University, Jinan, Shandong, China; ^7^ Laboratory Animal Center, Shandong University, Jinan, Shandong, China; ^8^ Central Laboratory, Shandong Provincial Hospital, Shandong University, Jinan, Shandong, China

**Keywords:** oocytes, ROS, IVM, metformin, aged mice

## Abstract

Human female fecundity decreases irreversibly as chronological age rises, adversely affecting oocyte quality, consequently worsening pregnancy outcomes and increasing the extent of birth defects. The first-line type 2 diabetes treatment metformin has been associated with delayed aging and reduction of oxidative stress; yet it remains unclear if metformin confers any benefits for oocytes from aged mice, particularly in the context of the assisted human reproductive technology (ART) known as *in vitro* maturation (IVM). Here, we found that adding metformin into the M16 culture medium of oocytes from aged mice significantly improved both oocyte maturation and early embryonic development. This study showed that metformin reduced the extent of meiotic defects and maintained a normal distribution of cortical granules (CGs). RNA-seq analysis of metformin-treated oocytes revealed genes apparently involved in the reduction of mitochondrial ROS. Further, the results supported that the metformin improved mitochondrial function, reduced apoptosis, increased the extent of autophagy, and reduced mitochondrial ROS *via* SIRT3-mediated acetylation status of SOD2K68 in oocytes from aged mice. Thus, this finding demonstrated a protective effect for metformin against the decreased quality of oocytes from aged mice to potentially improve ART success rates and illustrated a potential strategy to prevent or delay reproductive aging.

## Introduction

Female fecundity decreases as chronological age rises, with pronounced decreases after the age of 35 years in humans (Leridon, 2004). However, societal trends like the postponement of marriage and childbearing in women of reproductive age have increased the likelihood of infertility (Herbert et al., 2015). The parallel decline in both the quantity and quality of oocytes contributes to the gradual age-related decline in fertility (Djahanbakhch et al., 2007; Nelson et al., 2013), and at least 50% of the oocytes in women aged >40 are non-viable (Fu et al., 2014; Wang et al., 2021). Age-related declines in oocyte quality are associated with mitochondrial dysfunction, chromosome misalignment, and impaired spindle assembly (Eichenlaub-Ritter et al., 2004; Liu, 2016), leading to lower fertility rates, poor embryonic development, worsened pregnancy outcomes, and higher rates of birth defects (Herbert et al., 2015; Mikwar et al., 2020).

*In vitro* maturation (IVM) is a procedure wherein immature cumulus-oocyte complexes (COCs) are collected from small antral follicles and with the final stages of meiosis completed during *in vitro* culture (Wang et al., 2021). There are many small immature follicles by means of superovulation that using IVM can serve as embryo resources for *in vitro* fertilization (IVF), especially for advanced maternal age (Liu et al., 2018). Further, IVM offers substantial cost reductions compared to classic protocols for IVF, making it attractive for patients in developing countries (Coticchio et al., 2015). IVM research has progressed in recent years to significantly improve success rates and to provide evidence of safety in terms of neonatal and childhood outcomes: by 2015, more than 5000 IVM babies were born (Coticchio et al., 2015; Wakim et al., 2017). However, compared to conventional IVF, IVM results in a substantially lowered success rate and reduced oocyte developmental potentiality, and yet further IVM performance decreases have been associated with increased maternal age (Liu et al., 2018).

Age-related organ dysfunction is linked to disruption of redox homeostasis (Ruder et al., 2008), resulting from overproduction of reactive oxygen species (ROS) and/or deterioration of antioxidant defenses (Timoteo-Ferreira et al., 2021). Mitochondria are known to be the primary endogenous source of ROS (Hamatani et al., 2004; Liu et al., 2022), and an increase in ROS in oocytes has been shown to cause mitochondrial dysfunction and result in disrupted ATP production (Herbert et al., 2015; Marangos et al., 2015). Thus, it is plausible that scavenging ROS and reducing mitochondrial oxidative stress in oocytes could help alleviate age-related oocyte aging and fertility decline. To do this, researchers have explored supplementation of oocyte IVM medium with various antioxidant compounds, including quercetin, resveratrol, melatonin, coenzyme Q, and so on (Liu et al., 2018; Li et al., 2019a; Cao et al., 2020; Ma et al., 2020). The diabetes drug metformin has been shown to delay aging in several experimental models (Martin-Montalvo et al., 2013; Barzilai et al., 2016; Fang et al., 2018) and to reduce oxidative stress and germ cell loss (Ghasemnejad‐Berenji et al., 2018). During oocyte maturation, mitochondria supply the majority of the cellular ATP *via* the respiratory chain for the generation of cellular energy (Yu et al., 2010). Metformin has been shown to act *via* both AMP-activated protein kinase and inhibition of mitochondrial respiration (Rena et al., 2017), yet it remains unclear if metformin confers any benefits for oocytes from aged mice, particularly in the context of the assisted human reproductive technology (ART) known as IVM.

In this study, we explored the effects of metformin in promoting IVM and subsequent formation of blastocysts using aged mice models. The results showed that metformin can improve mitochondrial function, reduce meiotic defects, and this study supported a protective mechanism that metformin can reduce mitochondrial ROS *via* SIRT3-mediated acetylation status of SOD2K68 (SOD2K68ac) in oocytes from aged mice, suggesting the use of metformin as a potential strategy to reduce meiotic structure defects in oocytes from aged mice to potentially extend reproductive capacity.

## Results

### Treatment of oocytes from aged mice with metformin improves meiotic maturation and early embryonic development

**FIGURE 1:**
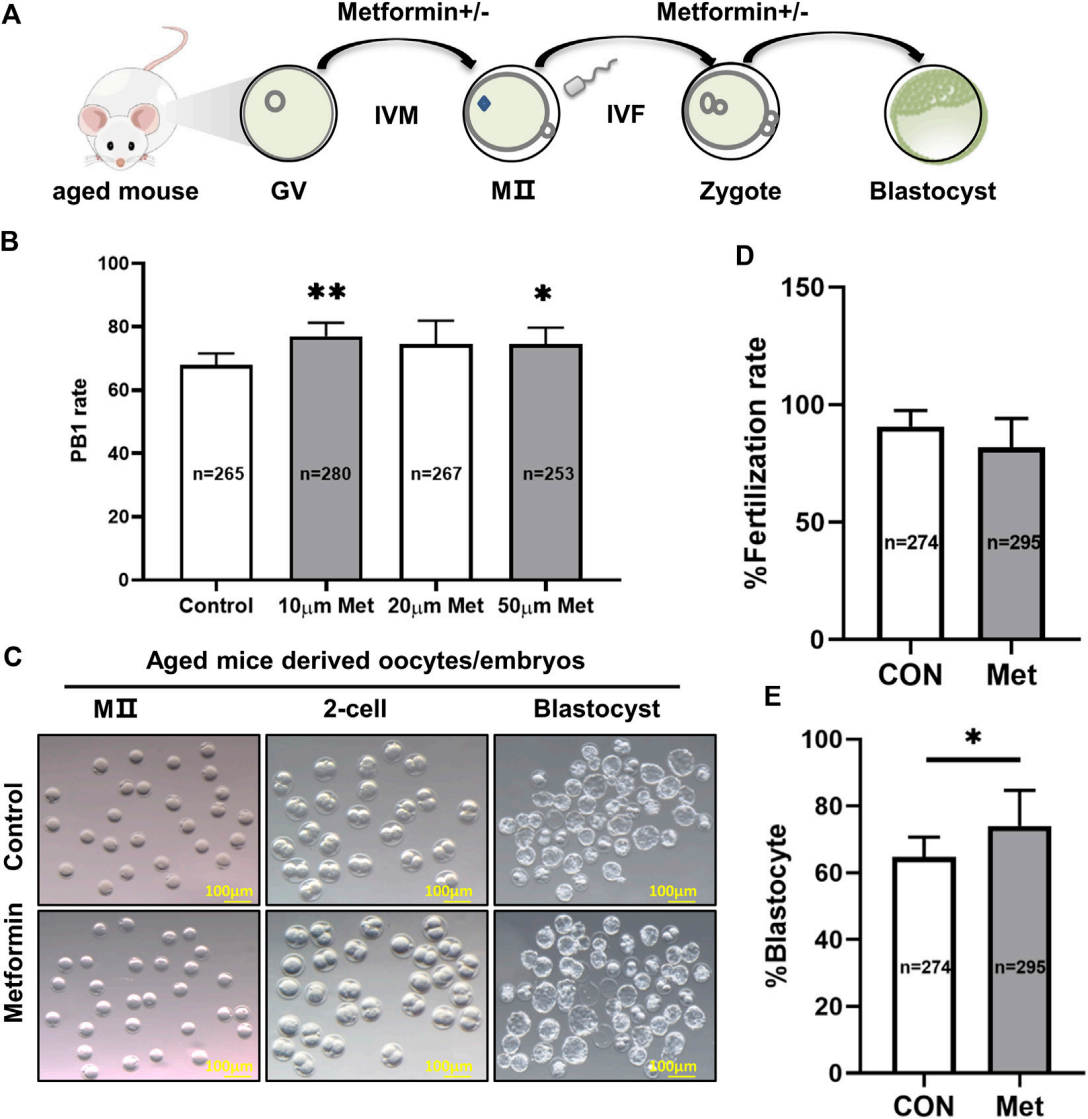
Treatment of oocytes from aged mice with metformin improves meiotic maturation and early embryonic development. **(A)** Schematic diagram showing the design for assessing metformin-treated oocytes from aged mice maturation and early embryonic development (up to the blastocyst stage) in the M16 medium *in vitro*. **(B)** Quantitative analysis of PB1 extrusion in the concentrations of 0 μM (*n* = 180 oocytes), 10 μM (*n* = 215), 20 μM (*n* = 199), and 50 μM (*n* = 188) metformin. **(C)** Representative images of oocytes and embryos cultured *in vitro* at the MII, 2-cell, and blastocyst stages. Scale bar, 100 μm. **(D)** Quantitative analysis of fertility rate with (*n* = 295) or without (*n* = 274) 10 μM metformin treatment. **(E)** Quantitative analysis of the blastocyst formation rate with (*n* = 295) or without (*n* = 274)10 μM metformin treatment. Data are means ± SD of at least three independent experiments. Means ± SD, *p < 0.05 vs. control as calculated by two-tailed unpaired Student’s t-tests, the % data are subjected to by two-tailed unpaired Student’s t-tests after an arcsine-square-root transformation.

To investigate whether metformin impacts oocytes development from aged mice, 1065 GV (germinal vesicle) -stage oocytes from 9- to 10-month-old female mice were collected and cultured in M16 medium with 10, 20, or 50 μM metformin or control for 16 h (Figure 1A). The 10 μM metformin significantly increased the rate of polar body (PB1) extrusion compared to the control (10 μM metformin = 77.0 ± 4.28%, *n* = 280 vs. control = 67.9 ± 3.60%, *n* = 265; *p* < 0.05); note that the 10 µM concentration outperformed the other treatments (10 μM metformin = 77.0 ± 4.28%, *n* = 280 vs. 20 μM metformin = 74.60 ± 7.36%, *n* = 267 vs. 50 μM metformin = 74.65 ± 5.03%, *n* = 253) (Figure 1B). These results clearly suggest that treating oocytes from aged mice with metformin can significantly promote the IVM rate, and we used a 10 μM metformin concentration for all subsequent experiments, because it was the lower concentration that increased PB1 rate with respect to the control.

We next assessed the potential involvement of metformin in early embryonic development from aged mice by performing IVF with or without 10 μM metformin. We found no significant differences in the rate of fertilization between the two groups (metformin = 81.86 ± 12.36%, *n* = 295 vs. control = 90.79 ± 6.72%, *n* = 274; *p* > 0.05). However, the presence of metformin in the culture medium significantly increased by 9.4% (metformin = 74.02 ± 10.71%, *n* = 295 vs. control = 64.7 ± 5.98%, *n* = 274; *p* < 0.05) which developed into blastocysts (*p* < 0.05) (Figure 1E). These results suggest that metformin treatment of oocytes from aged mice can apparently improve both IVM rates and early embryonic development.

### Metformin administration alleviates meiotic defects of oocytes and maintains a normal distribution of cortical granules from aged mice

**FIGURE 2:**
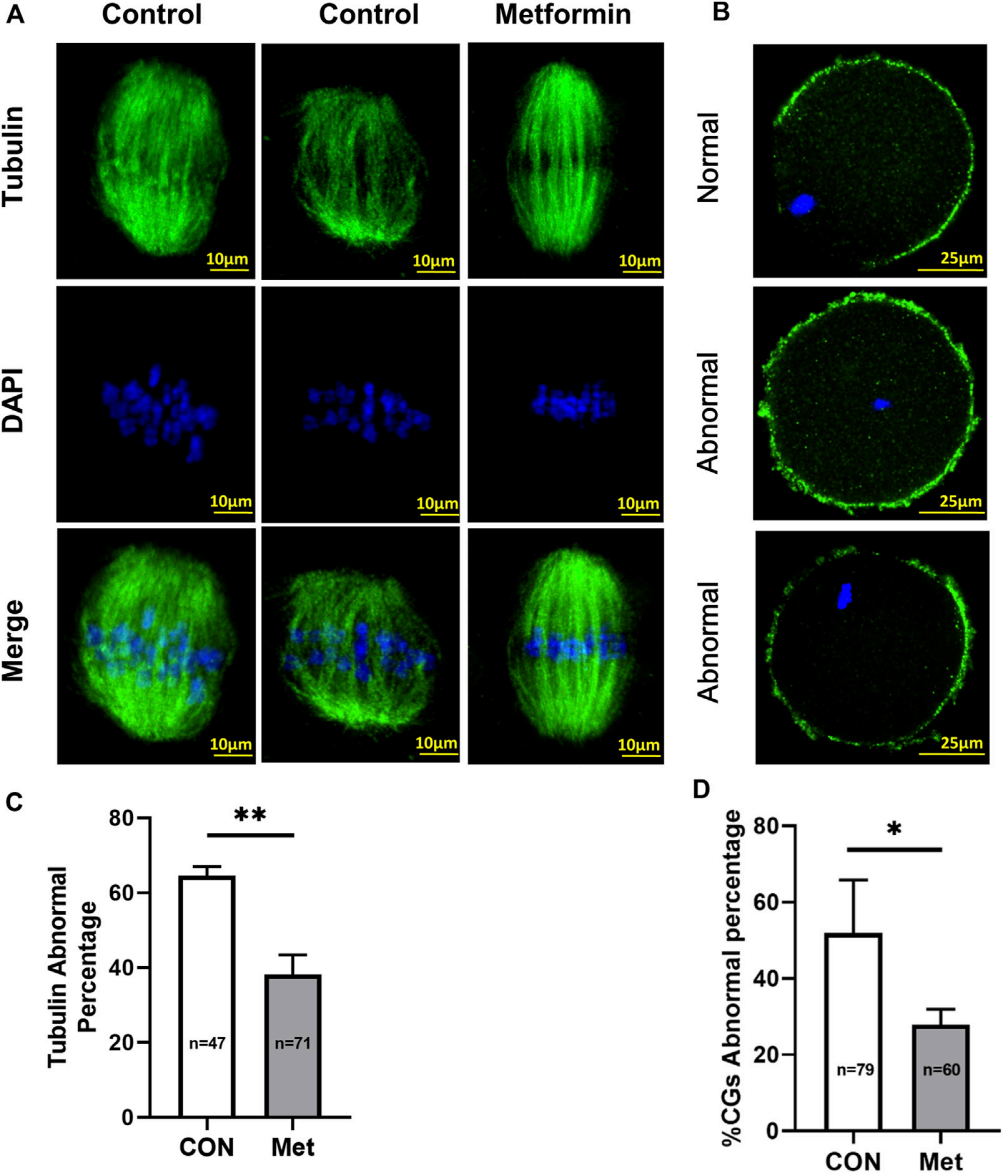
Metformin alleviates the meiotic defects of oocytes from aged mice and maintains a normal distribution of cortical granules (CGs).**(A)** Representative images of spindle/chromosome organization in MⅡ stage oocytes after *in vitro* maturation (IVM) oocytes from aged (7–9 month old) mice in the control and metformin-treatment groups. Spindles were stained with an α-tubulin antibody (green); chromosomes were counterstained with DAPI (blue). Scale bar, 10 μm. **(B)** Distinct distributions of cortical granules (CGs) in control vs. metformin-treated oocytes from aged mice. CGs in oocytes were stained with lens culinaris (LCA)-FITC (fluorescein isothiocyanate) (green); and chromosomes were counterstained with DAPI (blue). Scale bar, 25 μm. **(C)** Quantification of abnormal spindle/chromosomes oocytes from aged mice in the control (*n* = 47) and metformin-treatment group (*n* = 71). **(D)** The proportion of oocytes with abnormally distributed CGs with (*n* = 60) or without (*n* = 79) metformin treatment. Data are means ± SD of at least three independent experiments. Means ± SD, *p < 0.05, **p < 0.01 vs. control as calculated by two-tailed unpaired Student’s t-tests, the % data are subjected to by two-tailed unpaired Student’s t-tests after an arcsine-square-root transformation.

It has been widely reported that oocyte quality is determined by spindle morphology and chromosome alignment (Huang et al., 2015; Chen et al., 2016). Further, studies have reported that abnormal spindle morphology and aneuploidy in oocytes from aged mice can lead to decreased fertilization rates, increased risk of miscarriage, and birth defects in children (Martin-Montalvo et al., 2013; Barzilai et al., 2016; Cao et al., 2020). Our confocal microscopy analysis of MII-stage oocytes after IVM from aged mice revealed multiple spindle morphology and chromosome alignment abnormalities, including elongated spindles, an apparent lack of poles, and chromosome misalignment (Figure 2A). However, the metformin treatment group significantly reduced the proportion of abnormal chromosomes and spindles (metformin = 38.19 ± 5.24%, *n* = 71 vs. control = 64.60 ± 2.44%, *n* = 47; *p* < 0.01), as observing orderly aligned chromosomes on the equatorial plate and bipolar spindles (Figure 2C).

The CGs is regarded as an informative indicator of oocyte cytoplasmic maturation that can block polyspermy following fertilization (Walls and Hart, 2018). We assessed whether metformin affects the distribution of CGs of oocytes from aged mice using Lens culinaris agglutinin (LCA)-FITC staining and confocal microscopy. As the meiotic maturation is completed, normal distribution of CGs is distributed evenly in the oocyte subcortical region and except the CG-free domain near the chromosomes, whereas the abnormal distribution of CGs is discontinuous and weak signals in the oocyte cortex and without leaving CG-free domains. We found that the proportions of abnormal distribution of CGs were significantly reduced in the metformin treatment group, but more than 50% (metformin = 27.94 ± 4.01%, *n* = 60 vs. control = 51.98 ± 13.88%, *n* = 79; *p* < 0.05) lost this normal localization in the control group. (Figures 2B,D). Altogether, these results suggest that metformin may rescue the meiotic defects and recover the cytoplasmic maturation.

### Whole transcriptome analysis of metformin-treated oocytes from aged mice suggests that metformin mitigates oxidative stress

**FIGURE 3:**
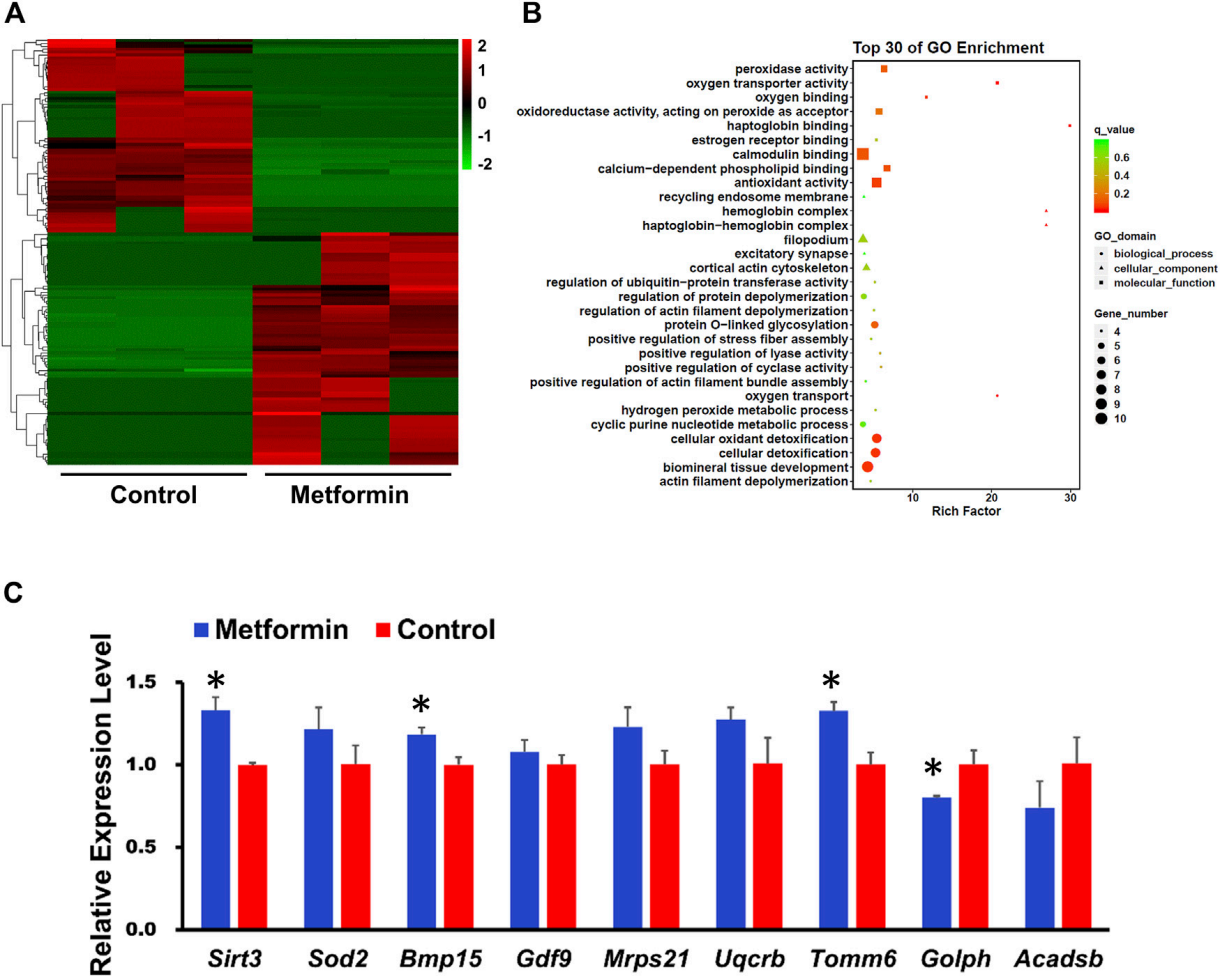
Whole-genome transcription analysis of oocyte after IVM from aged mice with or without metformin treatment. **(A)** Heatmap illustration showing differentially expressed transcripts in MⅡ stage oocytes after IVM with or without metformin treatment. **(B)** GO analysis of the top of 30 differentially expressed genes (DEGs) shows the biological processes affected by the altered mRNA expression by comparing the gene expression in MⅡ stage oocytes after IVM with or without metformin treatment. **(C)** qPCR results shows the expression levels of the indicated transcripts in control and metformin-treated oocytes. Data are means ± SD of at least three independent experiments. Means ± SD, *p < 0.05 vs. control as calculated by two-tailed unpaired Student’s t-tests.

We next performed a single-cell transcriptome analysis of oocytes from aged mice, including both metformin-treated and control samples. Compared with controls, the metformin-treated oocytes had 295 up-regulated differentially expressed genes (DEGs) and 155 down-regulated genes (with the following cutoff criterion: adjusted *p*-values less than 0.05 (Figure 3A). Gene Ontology (GO) analysis of the top-30-ranking DEGs revealed enrichment for functional annotations including oxidative phosphorylation, oxygen binding, oxidoreductase activity, antioxidant activity, oxygen transport, cellular oxidant detoxification, and hydrogen peroxide metabolic process (Figure 3B). The expression trend data for 11 randomly selected genes in each group was verified using qPCR (Figure 3C). Numerous up-regulated genes have annotated functions related to anti-oxidative metabolism (*Sod2*), oocyte and embryonic development (*Bmp15, Gdf9*), autophagy (*Tomm6*), aging (*Sirt3*), and the mitochondrial function (*Uqcrb, Mrps21*). Some of the down-regulated genes had annotated functions concerning lipid binding (*Golph3l*) and metabolic processes (*Acadsb*) (Figure 3C). Our transcriptome analysis of MII-stage oocytes thus indicates that metformin treatment can mitigate oxidative stress in oocytes from aged mice.

### Metformin reduces ROS and improves mitochondrial function in oocytes from aged mice

**FIGURE 4:**
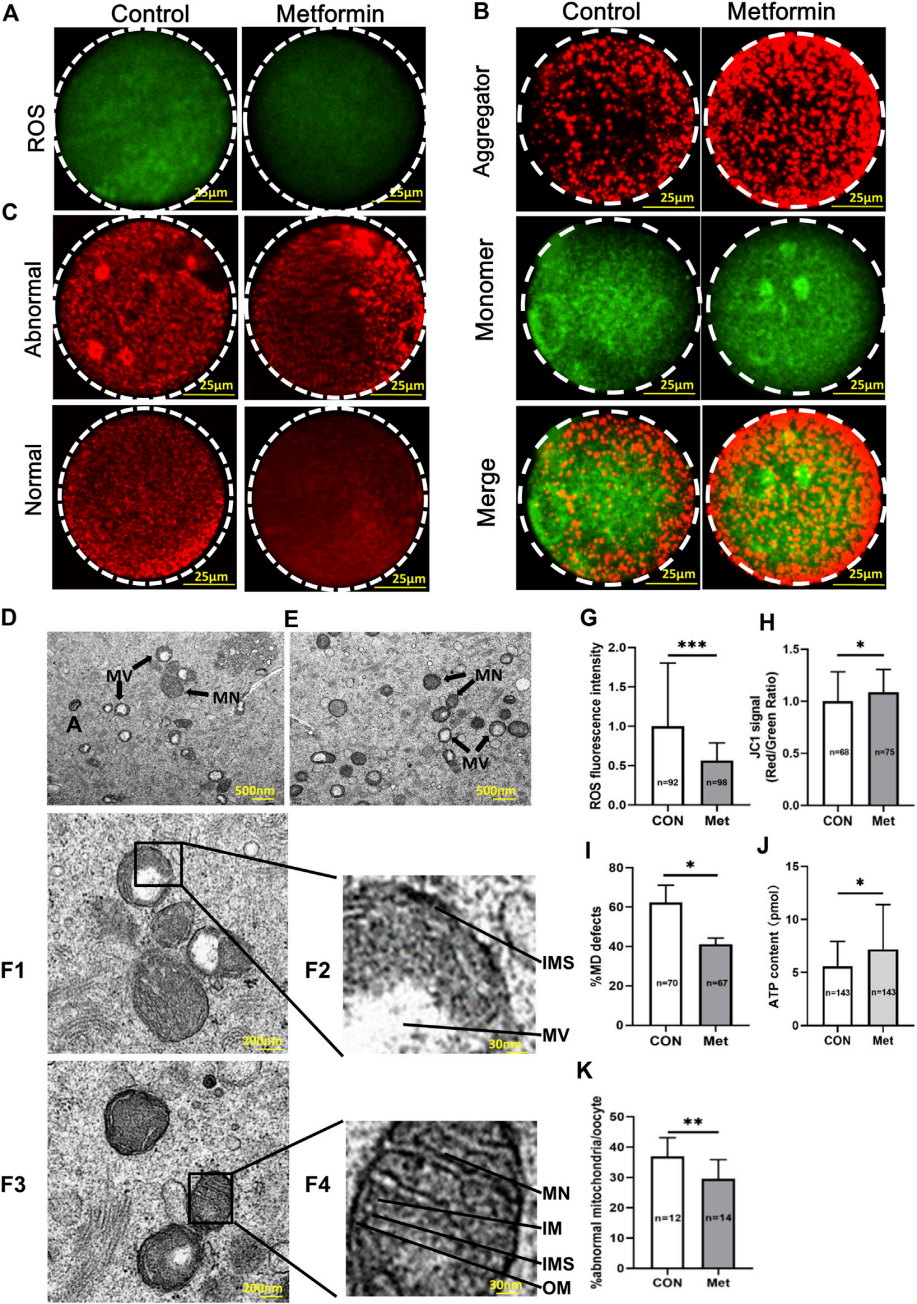
Metformin reduces ROS, attenuates mitochondrial function, and improves mitochondrial ultrastructure of oocytes from aged mice. **(A)** Representative images of staining for ROS using carboxy-H2DCF diacetate fluorescence (green) in MⅡ stage oocytes after IVM with or without metformin treatment. Scale bar, 100 μm. **(B)** JC-1 staining shows the mitochondrial membrane potential (MMP), for oocytes treated in control and metformin-treated oocytes. Note that the same oocytes were observed in both the TRITC channel (red fluorescence) and the FITC channel (green fluorescence). Scale bar, 25 μm. **(C)** Distribution of mitochondria, as stained using MitoTracker-Red in oocytes treated with or without metformin. Scale bar, 25 μm. **(D)** Representative transmission electron microscopy (TEM) micrographs of mitochondria from control oocytes. Scale bar = 500 nm. Note the normal (Mn) and vacuolated (Mv) mitochondria. **(E)** Representative TEM micrographs of mitochondria from metformin-treated oocytes. **(F1)** Representative electron micrograph of mitochondria of the control group oocytes. Scale bar, 200 nm. **(F2)** Higher magnification views of boxed regions in **(F1)**, highlighting abnormal mitochondrial ultrastructural features, including the mitochondrial vacuole (MV), a narrowed inter-membrane space (IMS), and myelin figures (MF). Scale bar, 30 nm. **(F3)** Representative TEM micrographs of mitochondria from an oocyte from the metformin treatment group. Scale bar, 200 nm. **(F4)** Higher magnification view of the boxed region from F3, highlighting a vacuolated mitochondrion, showing mitochondrial cristae (MC), the structures of the outer membrane (OM), and inner membrane (IM), as well as a well-defined inter-membrane space (IMS). Scale bar, 30 nm. **(G)** Quantification of ROS fluorescence in control (*n* = 92) and metformin-treated oocytes (*n* = 98). **(H)** Quantification of MMP (red/green fluorescence intensity ratio) upon staining control oocytes (*n* = 68) and metformin-treated oocytes (*n* = 75) with JC-1. **(I)** Quantification of the proportion of oocytes with abnormally distributed mitochondria in oocytes treated with (*n* = 67) or without metformin (*n* = 70). **(J)** Quantification of the intra-oocyte adenosine triphosphate (ATP) content in control (*n* = 143) and metformin-treated oocytes (*n* = 143). **(K)** Quantification of abnormal mitochondrial ultrastructural features in MⅡ stage oocytes after IVM with (*n* = 14) or without metformin treatment (*n* = 12). Data are means ± SD of at least three independent experiments. Means ± SD, *p < 0.05, ***p < 0.001 vs. control as calculated by two-tailed unpaired Student’s t-tests, the % data are subjected to by two-tailed unpaired Student’s t-tests after an arcsine-square-root transformation.

ROS is one of the causes of age-related decline in fertility (Appasamy et al., 2008; Igarashi et al., 2015; Becatti et al., 2018), with specific reports of dysregulated mitochondrial redox balance, destabilization of mitochondrial DNA, as well as disrupted oocyte membrane function and deterioration (Droge, 2002; Tamura et al., 2020). We used carboxy-H2DCF diacetate to stain oocytes from aged mice with or without metformin treatment to evaluate whether metformin impacts ROS. Briefly, we found that the ROS level was significantly reduced in the metformin treatment oocytes compared to controls (Figures 4A,G; metformin = 31.78 ± 12.57, *n* = 98 vs. control = 56.22 ± 45.12, *n* = 92; *p* < 0.001).

Mitochondria are considered the major site of intracellular ROS production, as more than 90% of total cellular oxygen reduction involves electron carriers of the mitochondrial respiratory chain (Hamatani et al., 2004; Nohl et al., 2005). Given that previous studies of oocytes from aged mice have revealed mitochondrial dysfunctions such as defects in mitochondrial membrane potential (MMP), mitochondrial distribution (MD), and ATP levels (Zeng et al., 2018; Pasquariello et al., 2019; Cao et al., 2020), we focused our assessments of mitochondrial function on these aspects.

Pursing the idea that metformin may provide beneficial effect(s) against age-induced mitochondrial dysfunction of oocytes, we investigated the mitochondrial membrane potential (MMP) using a standard immunolabeling protocol with JC-1 wherein increasing membrane potential induces a green-to-red shift (Muhammad et al., 2020). This quantitative analysis confirmed that the red/green ratio was significantly increased in the metformin-treated oocytes compared to controls (Figures 4B,H; metformin = 1.09 ± 0.22, *n* = 75 vs. control = 1.0 ± 0.28, *n* = 68; *p* < 0.05). To further assess mitochondrial function, we used MitoTracker-Red staining to examine the mitochondrial distribution of oocytes from aged mice. Confocal microscopy showed that whereas around 60% (metformin = 41.15 ± 3.18%, *n* = 67 vs. control = 62.41 ± 8.72%, *n* = 70) of the oocytes from aged mice without metformin treatment had abnormally distributed mitochondria (*i.e.*, asymmetric clustering), this proportion was significantly decreased by that given metformin treatment (Figures 4C,I). The distribution of mitochondria is known to dynamically impact ATP homeostasis, and mitochondrial disorders frequently lead to reduced ATP production (Wakim et al., 2017). Our analysis showed higher ATP levels in metformin-treated oocytes than in untreated controls (Figure 4J; metformin = 7.18 ± 4.21, *n* = 143 vs. control = 5.57 ± 2.36, *n* = 143; *p* < 0.001). Together, these results show that metformin treatment of oocytes from aged mice mitigates oxidative stress, specifically by reducing ROS levels and promoting mitochondrial function.

### Metformin improves the mitochondrial ultrastructure of oocytes from aged mice

Given our observations of disrupted mitochondrial distribution, ATP production and MMP, we next evaluated whether metformin treatment influences mitochondrial ultrastructure in oocytes from aged mice. Our transmission electron microscopy (TEM) analysis showed that the metformin-treated oocytes had a significantly reduced extent of abnormal mitochondrial ultrastructure phenotypes (Figure 4K; metformin = 29.52 ± 6.33%, *n* = 14 vs. control = 36.92 ± 6.20%, *n* = 12; *p* < 0.01), which typically exhibit blurred cristae, narrowed inter-membrane spaces (Figures 4D, F1, F2). The normal mitochondrial ultrastructure had defined cristae, clearly visible intact inner and outer membranes, and well-defined intermembrane spaces (Figures 4E, F3, F4). Collectively, these results demonstrate that metformin can somehow improve age-related abnormal ultrastructure phenotypes of oocyte mitochondria.

### Metformin decreases apoptosis and increases autophagy of oocytes from aged mice

**FIGURE 5:**
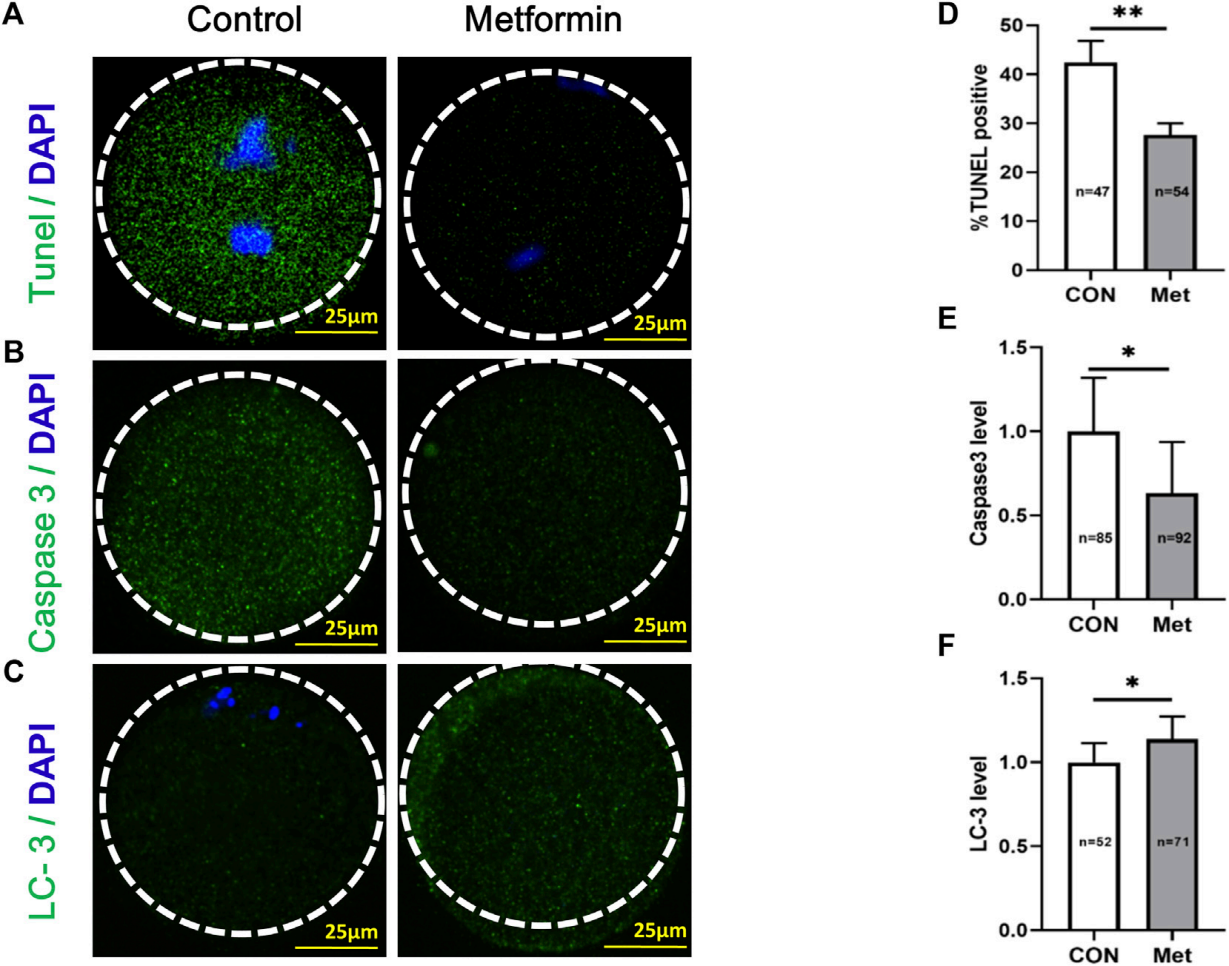
Metformin decreases apoptosis and increases anti-oxidative activity in oocytes from aged mice. **(A)** Representative apoptosis immunofluorescence images from TUNEL assays in MⅡ stage oocytes after IVM with or without metformin treatment. The green fluorescence indicates TUNEL-positive oocytes; chromosomes were counterstained with DAPI (blue). Scale bar, 25 μm. **(B)** Representative apoptosis immunofluorescence images of levels of activated Caspase 3 in MⅡ stage oocytes after IVM with or without metformin treatment. Scale bar, 25 μm. **(C)** Representative immunofluorescence images of autophagosomes (LC3-II puncta) in MⅡ stage oocytes after IVM with or without metformin treatment. Scale bar, 25 μm. **(D)** The percentage of TUNEL-positive oocytes in control (*n* = 47) and metformin-treated oocytes (*n* = 54). **(E)** Quantification of Caspase 3 intensity in control (*n* = 85) and metformin-treated oocytes (*n* = 92). **(F)** Quantitative analysis of fluorescence intensity showing the LC-3 level in control (*n* = 52) and metformin-treated oocytes (*n* = 71). Data are means ± SD of at least three independent experiments. Means ± SD, *p < 0.05, **p < 0.01 vs. control as calculated by two-tailed unpaired Student’s t-tests, the % data are subjected to by two-tailed unpaired Student’s t-tests after an arcsine-square-root transformation.

Given the known impacts of oxidative stress in the induction of apoptosis (Chen et al., 2014; Dimozi et al., 2015), we next performed a TUNEL (Terminal deoxynucleotidyl transferase dUTP nick-end labeling) analysis to investigate potential impacts(s) of metformin on oocytes from aged mice. There were significantly fewer apoptotic oocytes in the metformin-treated group compared to the untreated controls (Figures 5A,D; metformin = 27.66 ± 2.34%, *n* = 54 vs. control = 42.48 ± 4.39%, *n* = 47; *p* < 0.01). Further, we found that the extent of caspase3 activation was significantly reduced in the metformin-treated group compared to the untreated controls (Figures 5B,E; metformin = 0.63 ± 0.30, *n* = 92 vs. control = 1.0 ± 0.32, *n* = 85; *p* < 0.05). Previous studies have shown that, during follicular development, oocytes block the induction of apoptosis by employing autophagy as a “survival mechanism” (Pepling, 2012). We conducted immunofluorescence staining of oocytes from aged mice against the autophagy marker protein LC3 (Microtubule-associated protein light chain 3) and found that metformin treatment significantly increased LC3 levels (Figures 5C,F; metformin = 1.14 ± 0.13, *n* = 71 vs. control = 1.0 ± 0.11, *n* = 52; *p* < 0.05). Thus, metformin treatment can both increase autophagy activity and decrease the extent of apoptosis in oocytes from aged mice.

### Metformin treatment can mitigate ROS *via* SIRT3-mediated reduction of SOD2K68 acetylation in oocytes from aged mice

**FIGURE 6:**
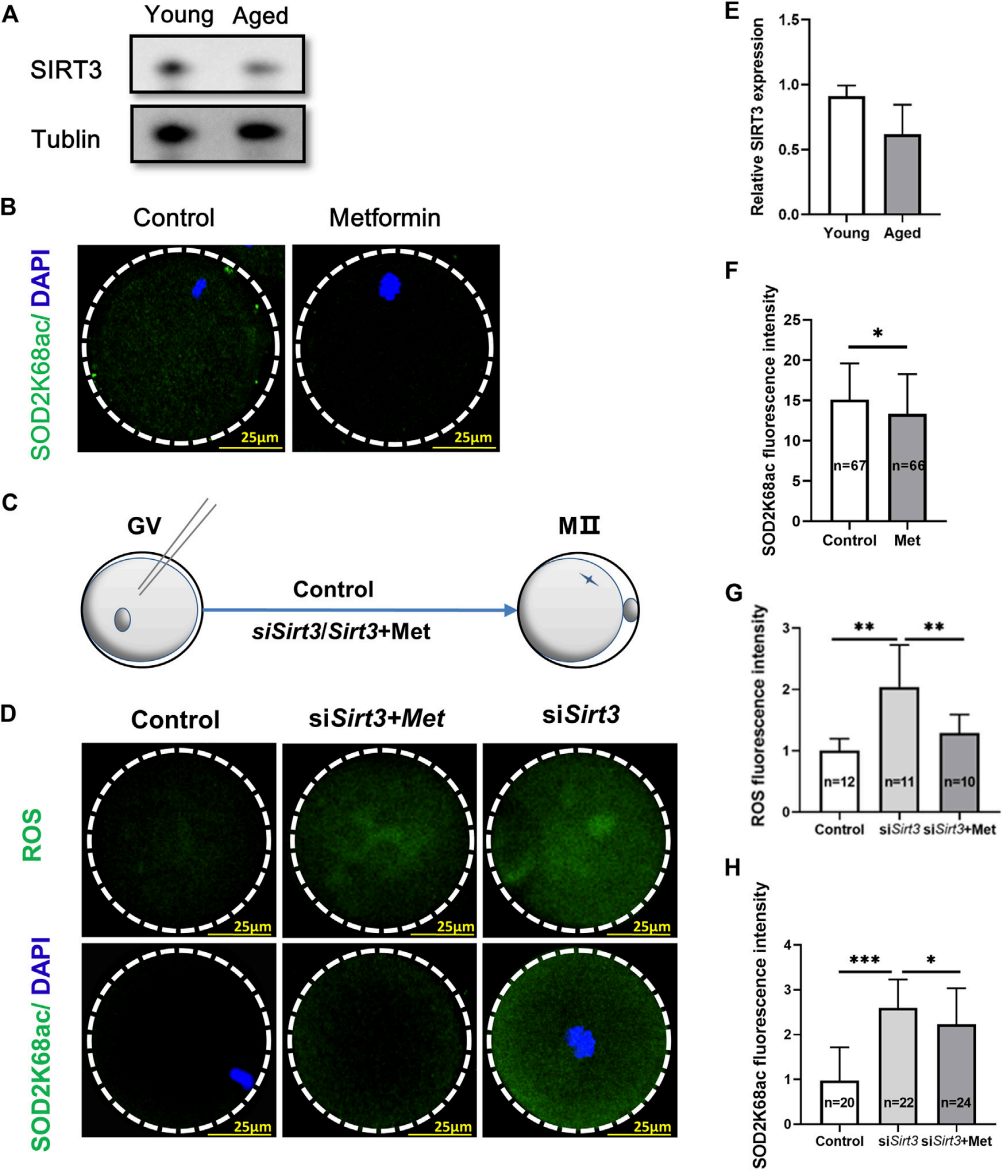
Oocytes from aged mice have reduced SIRT3 levels and metformin treatment can mitigate SIRT3-induced SOD2K68ac in oocytes from aged mice. **(A)** Immunoblotting showing reduced SIRT3 expression in GV stage oocytes after IVM from aged mice (42–45 weeks) compare with young (6–8 weeks) mice. Tublin was used as a loading control. **(B)** Representative immunofluorescence images of SOD2K68ac in MⅡ stage oocytes after IVM with or without metformin treatment. Scale bar, 25 μm. **(C)** Schematic illustration of the experiment to assess whether metformin treatment of oocytes from aged mice that were injected with a Sirt3-targeting siRNA affects SOD2K68ac levels. **(D)** Metformin lowers the acetylation levels of SOD2K68 and ROS upon the reduction of SIRT3 expression by comparing the injected control (PBS), siSirt3 (injected with Sirt3 siRNA), and siSirt3 + metformin treatment oocytes from aged mice. Scale bar, 25 μm. **(E)** Quantification of the data from panel A; the samples comprised 100 oocytes from the young and old mice. **(F)** Quantification of the data from panel B; the samples comprised 100 oocytes from the young and old mice. **(G,H)** Quantitative analysis of the data from panel D. Data are means ± SD of at least three independent experiments. Means ± SD, *p < 0.05, **p < 0.01, ***p < 0.001 vs. control as calculated by two-tailed unpaired Student’s t-tests.

SIRT3 (Sirtuin 3) is localized in the mitochondria and regulates the acetylation status of the most mitochondrial proteins (Sultana et al., 2016), often associated with aging (Ansari et al., 2017). Previous studies have shown that SIRT3 can deacetylate SOD2K68, consequently activating SOD2, thereby increasing its mitochondrial anti-oxidative activity (Someya et al., 2010; Liu et al., 2017; Cao et al., 2020), and acetylation levels of SOD2 are negatively associated with its enzymatic activity (Cao et al., 2020). However, whether metformin can mitigate mitochondrial ROS by SIRT3-mediated reduction of SOD2K68ac in IVM from oocytes from aged mice still needs to be investigated. We next assessed whether maternal age affects SIRT3 level in GV oocytes, immunoblotting of oocytes from old (42–45 weeks) and young mice (6–8 weeks) revealed that oocytes from aged mice had reduced SIRT3 expression compared with that in young mice (Figures 6A,E; young = 0.91 ± 0.08, *n* = 3 vs. aged = 0.62 ± 0.23, *n* = 3; *p* = 0.11). We next conducted SOD2K68ac staining using confocal microscopy in the metformin-treated group and the untreated controls from the MII-stage oocytes after IVM, and found that metformin-treated significantly reduced SOD2K68ac levels (Figures 6B,F; metformin = 13.35 ± 4.90, *n* = 66 vs. control = 15.11 ± 4.48, *n* = 67; *p* < 0.05). We further examined whether metformin in the IVM culture medium of oocytes from aged mice may affect the SOD2K68ac by regulating SIRT3, consequently reducing mitochondrial ROS in oocytes from aged mice. Fully grown GV oocytes from old mice were injected with exogenous *Sirt3* siRNAs or PBS, as the negative control (Figure 6C).

Monitoring ROS levels in live oocytes using carboxy-H2DCF diacetate fluorescent dye with confocal microscopy showed that the ROS accumulation was significantly increased in the si*Sirt3* oocytes compared with control (PBS) oocytes. Moreover, treatment of si*Sirt3* oocytes with metformin led to a significant reduction in ROS as compared with the untreated si*Sirt3* oocytes (Figures 6D,G; si*Sirt3* = 2.04 ± 0.68, *n* = 11 vs. control = 1.0 ± 0.19, *n* = 12; *p* < 0.01; si*Sirt3* = 2.04 ± 0.68, *n* = 11 vs. si*Sirt3+*metformin = 1.29 ± 0.29, *n* = 10; *p* < 0.01). Similarly, assessment of SOD2K68ac levels showed that siSirt3 oocytes had significantly increased SOD2K68ac levels compared to the control cells, and also showed that metformin treatment could partially rescue this increased SOD2K68ac phenotype (Figures 6D,H; si*Sirt3* = 2.60 ± 0.63, *n* = 22 vs. control = 0.97 ± 0.74, *n* = 20; *p* < 0.001; si*Sirt3* = 2.60 ± 0.63, *n* = 22 vs. si*Sirt3+*metformin = 2.23 ± 0.81, *n* = 24; *p* < 0.05). Thus, metformin can mitigate mitochondrial ROS *via* SIRT3-induced reduction of SOD2K68ac in oocytes from aged mice.

## Discussion

For human ART, improved IVM culture conditions enable some immature oocytes to develop into embryos. Our results in the present study confirm that metformin can improve IVM success rates in oocytes from aged mice. We demonstrated improvement of early embryonic development upon metformin treatment, provided insights about how metformin impacts the meiotic defects of oocytes and contributes to maintaining a normal distribution of cortical granules in oocytes from aged mice. Our results support that metformin improved the quality of oocytes from aged mice by recovering mitochondrial function, which, in turn, reducing the accumulated ROS suppressing apoptosis and promoting autophagy during aging, we show that metformin can mitigate ROS *via* SIRT3-mediated reduction of SOD2K68ac in oocytes from aged mice.

Biological aging primes the development of age-related diseases (López-Otín et al., 2013). The ovary is the first organ to show signs of biological aging in women (usually occurring between the ages of 40 and 58 years), and seriously affects women’s health and quality of life (Fritz and Jindal, 2018; Schach et al., 2021). More than 90% of age-related deterioration of embryo competence is caused by aneuploidy, which arises principally from chromosomal missegregation in meiosis (Song et al., 2016). Calorie restriction (CR) can improve health conditions, increase lifespan, and the extension of maternal fertility (Selesniemi et al., 2011; Zhang et al., 2021), but it has also been reported to cause reduced fertility or even complete infertility (Tilly and Sinclair, 2013; Dou et al., 2017). Previous studies have shown that metformin can repair quality defects of oocytes induced by arecoline or dehydroepiandrosterone in mice (Huang et al., 2015; Li et al., 2020). The present study showed that metformin can attenuate age-related anomalous spindle formation and chromosome alignment in the nucleus and can also help maintain a normal distribution of CGs in the cytoplasm of oocytes. Clinically, improved IVM culture conditions can offer an effective approach to acquiring more high-quality oocytes for ART to improve the fertility of aged females (Miao et al., 2020). Further, IVM has been used to treat patients with a range of fertility-related conditions including fertility preservation, thus reducing the risk of thrombotic events in at-risk patients and those diagnosed with FSH-resistant ovaries (Walls and Hart, 2018). The asynchronous maturation of the cytoplasm and nucleus of the oocyte is a major challenge when seeking to improve the quality of oocytes in IVM, and emerging evidence has suggested that metformin can help to overcome this (Coticchio et al., 2015). We found no significant differences in the rate of fertilization after metformin treatment. However, we observed that metformin improved IVM rate and early embryonic development of oocytes in naturally aging mice (in which fertility is known to decline rapidly). Thus, the present study supports that metformin has potential clinical benefits for women who wish to postpone childbearing, provided a conceptual basis for improving IVM culture conditions and using immature oocytes from aged humans.

ROS fundamental in mediating folliculogenesis, meiosis, ovulation, and embryonic development as secondary messengers for cellular signaling (Agarwal et al., 2012), but overabundant ROS by *in vitro* culture, post-ovulatory aging and other factors will result in oxidative stress and contributors to aging (Finkel and Holbrook, 2000; Lin et al., 2018). Mitochondria are a major site of ROS damage, and are the known induction site for the intrinsic induction pathway of autophagy (Lyamzaev et al., 2018). Mitochondria generate ATP *via* oxidative metabolism, and mitochondrial activity can be used to assess the quality of oocytes. Previous studies have established requirements for a low ROS index and for a relatively high ATP level in oocytes (Muhammad et al., 2020), and both ATP metabolism and ROS are directly associated with mitochondrial functional which can be assessed through distribution of mitochondria, MMP, ATP level, mitochondrial ultrastructure and gene expression (Zhang et al., 2006; Cao et al., 2020). The increased mitochondrial dysfunction and reduced autophagy can activate the mitochondrial-related apoptotic signaling pathway during the maturation of oocytes (Tatone et al., 2006). Metformin can reduce oxidative stress, increase antioxidant defenses, increased autophagy and reduce the extent of chronic inflammation (Martin-Montalvo et al., 2013; Bharath et al., 2020). Previous studies have shown that metformin can improve oocyte quality and embryo development and reverse ovulation dysfunction in polycystic ovary syndrome mice model by reducing ROS and improving mitochondrial function in oocytes (Huang et al., 2015), and can reduce apoptosis in blastocysts of obese mice (Louden et al., 2014). Emerging evidence strongly suggests that metformin improves the quality of oocytes from aged mice and subsequently promoting both oocyte maturation and early embryonic development in aged mice oocytes by recovering mitochondrial function, which, in turn, reduces the accumulation of ROS to suppress apoptosis and promote autophagy during aging. Our findings are in agreement with studies reporting that metformin protects against apoptosis and senescence in nucleus pulposus cells (Chen et al., 2016). Thus, this work supports that metformin can be used as an effective agent to prevent age-related mitochondrial dysfunction of the oocyte to alleviate the accumulation of excessive ROS levels.

Oxidative damage accelerates mouse ovarian aging by decreasing the expression of antioxidant genes (Lim and Luderer, 2011). *Sirt3* is an antiaging gene, the main deacetylase that inhibits mitochondrial ROS, and *Sirt3* participates in multiple signaling events including regulation of oxidative stress, mitochondrial biogenesis, apoptosis, and metabolic activity (Sultana et al., 2016). Mitochondrial SOD2 is an antioxidant enzyme and plays a crucial role in controlling ROS production (Chen et al., 2011). Emerging evidence shows that SIRT3/SOD2 signaling activation prevents oxidative stress and mitochondrial damage in multiple pathological and pathological conditions (Li et al., 2019b; Cao et al., 2020; Liu et al., 2021), and mitochondrial-ROS stimulated autophagic cell death dependent on the SIRT3/SOD2 pathway (Zhou et al., 2021). Our data confirm previous reports that SIRT3 levels are increased in oocytes from aged mice compared to young animals. Further, we show that metformin can ameliorate the mitochondrial-ROS after knockdown SIRT3 in oocytes from aged mice, leading to SOD2 deacetylation and activation, and yet SIRT3 can help eliminate ROS by transforming SOD2K68ac into SOD2. Hence our study represents a potential strategy for the treatment of reproductive aging.

In this study, we discovered that metformin treatment of oocytes from aged mice can attenuate age-related mitochondrial oxidative stress, alleviate meiotic defects, help maintain a normal distribution of CGs, promote oocyte maturation, and promote early embryonic development. We showed that metformin’s effects involved reduced apoptosis, increased extent of autophagy, and reduced mitochondrial-ROS, and demonstrated that metformin treatment can mitigate ROS *via* SIRT3-mediated reduction of SOD2K68ac in oocytes from aged mice. Thus, metformin represents a promising direction for future clinical applications in preventing reproductive aging and may help efforts to develop and improve IVM culture systems for patients of advanced maternal age. However, it needs to be emphasized that the use of metformin requires rigorous safety evaluations and careful monitoring for unanticipated impacts on pregnancy outcomes.

## Materials and methods

The protocol for this study was reviewed and approved by the Institutional Review Board of Reproductive Medicine, Shandong University [(2021) Ethical Review #33]. Unless otherwise noted, all chemicals and reagents used were purchased from the Sigma Chemical Company (St. Louis, MO, United States).

### Mice

Old ICR (Institute of Cancer Research) female mice from 42- to 45-week-old (when fertility declines rapidly) and young ICR female mice from 6- to 8-week-old were purchased from Beijing Vital River Experimental Animals Centre (Beijing, China). Mice were housed under a 12-h light: 12-h darkness cycle, in a controlled temperature and humidity animal facility, and were provided with water *ad libitum* and food.

### Oocyte collection and culture

To retrieve fully grown GV oocytes, mice were injected intraperitoneally with 10 IU Pregnant Mares Serum Gonadotropin (PMSG) (Ningbo Hormone Product Company, China). After 46–48 h, mice were ultimately sacrificed, cumulus-oocyte complexes were collected by manual rupturing of antral ovarian follicles, cumulus cells were removed by repeatedly pipetting. For IVM, denuded GV oocytes cultured were cultured in the small drops in M16 medium supplemented with or without different concentrations of metformin (10, 20, or 50 μM), subsequently covered with mineral oil were incubated under 6% CO_2_, 5% O_2_, and 90% N_2_ at 37°C for 16 h to determine the PB1 extrusion rate.

For IVF, mice were intraperitoneally injected with 10 IU PMSG, followed 48 h later by 10 IU of human chorionic gonadotrophin (hCG) (Ningbo Hormone Product Company, China). After 16 h, mice were ultimately sacrificed, oviductal ampullae were broken to release cumulus-oocyte complexes. Meanwhile, the adult male mice were sacrificed, and sperm was obtained from cauda epididymis *via* orchidectomy. Subsequently cumulus-oocyte complexes fertilized with adult male sperm (the concentration of sperm was (1–2.5) × 10^6^/ml) in G-IVF medium (Vitrolife, Sweden) supplemented with or without 10 μM metformin. Zygotes covered with mineral oil were incubated under 6% CO_2_, 5% O_2_, and 90% N_2_ at 37°C in G1 medium (Vitrolife, Sweden) to observe embryonic developmental potential with a stereomicroscope (Nikon SMZ1500).

### Oocyte microinjection

For microinjection in knockdown experiments, 10 pL *Sirt3*-targeting siRNA (10 ng/μL) was injected into GV stage oocytes, and the same amount of RNase-free PBS was injected as a control. Oocytes were arrested at the GV stage in M16 medium containing 2.5 mM milrinone for 20 h to promote the synthesis of new protein. Then, oocytes were cultured in the M16 medium supplemented with (siRNA + metformin) or without (siRNA) 10 μM metformin for 16 h. RNA was obtained from RiboBio (Guangzhou, China) and the sequences used are listed in Supplementary Table S2.

### ROS assessment

For measurement of intracellular ROS levels, oocytes were incubated in M2 medium containing 10 mM carboxy-H2DCF diacetate (Beyotime, China) for 30 min at 37°C. After being washed 3 times in an M2 medium and subsequently mounted on glass slides, oocytes were imaged with a confocal microscope (Dragonfly, Andor Technology, United Kingdom). The fluorescence intensity for each oocyte was measured with ImageJ (National Institutes of Health, United States).

### Measurement of ATP content

ATP measurement was performed using the luciferin–luciferase reaction (Bioluminescent Somatic Cell Assay Kit, Sigma, United States). Firstly, mixed for 5 s before detection, a linear regression of standard curve containing 11 ATP concentrations from 10 fmol to 10 pmol was used to determine oocyte ATP content. Then, ATP concentrations of oocytes were measured on an EnSpire Multimode Plate Reader (PerkinElmer, United States).

### Immunofluorescence microscopy

Immunofluorescence was performed as described previously (Cao et al., 2020). In brief, oocytes were fixed for 30 min with 4% paraformaldehyde, permeabilized for 20 min with 0.1% Triton X-100, and blocked with 1% BSA for 0.5 h. Samples were incubated with primary antibodies in 1% BSA for 1 h at room temperature. The primary antibodies were as follows: anti-active caspase-3 (Abcam, United States), anti-LC3 (Cell Signaling, United States), and anti-SIRT4 (Abcam, United States). After being washed three times for 5 min, samples were incubated with suitable secondary antibodies at room temperature for 1 h. To visualize the spindle, oocytes were probed with an anti-α-tubulin antibody (Sigma, United States). For the distribution of cortical granules staining, oocytes were probed with labeled lens culinaris agglutinin (Vectorlabs, United States). For the TUNEL staining, oocytes were probed with an *in situ* cell death kit (Roche, Swiss). To evaluate the mitochondrial membrane potential (MMP), oocytes were incubated in 2 μM JC-1 (Invitrogen, United States) for 30 min at 37°C. To detect the mitochondrial distribution, oocytes were incubated in 200 nM MitoTracker-Red (Invitrogen, United States) for 30 min at 37°C. To visualize chromosomes, oocytes were probed with DAPI (Solarbio, China) for 10 min. Oocytes were examined under a laser scanning confocal microscope (Dragonfly, Andor Technology, United Kingdom). Images were acquired by using the same confocal microscope settings within and between experiments. The mean fluorescence intensity of each oocyte was measured with ImageJ (National Institutes of Health, United States). The antibodies used in these experiments are shown in Supplementary Table S1.

### RNA sequencing and qPCR

For RNA sequencing, 15 MII stage oocytes after IVM from three mice were considered one group, and three replicates were assessed per group. The sample library was built with a smart-seq HT Kit (Takara, Japan) at Shanghai Sinomics Corporation and sequenced with an Illumina NovaSeq 6000 instrument (Illumina, United States). Raw data files are publicly available from the Gene Expression Omnibus (GEO) database under accession number GSE201098. Total RNA was extracted from samples using RNeasy Mini Kits (Qiagen, Germany) following the manufacturer’s protocol. Expression levels of mRNA were partially verified by qPCR experiments performed with a Light Cycler 480 (Roche, Swiss). The mRNA levels were normalized to endogenous GAPDH (Glyceraldehyde-3-phosphate dehydrogenase) mRNA levels using calculations performed with Microsoft Excel. Primer sequences are shown in Supplementary Table S2.

### Electron microscopy

Oocytes from five mice treated with or without metformin were used for each sample. To analyze mitochondrial ultrastructure, oocytes were prepared for transmission electron microscopy (TEM). Morphometric analysis of mitochondrial ultrastructure was based on electron micrographs at 30,000-fold magnification.

### Western blot analysis

For total protein extraction, a total of 100 oocytes were lysed in SDS buffer by boiling for 5 min. The sample was separated by 10% SDS-PAGE and transferred to a PVDF membrane (Bio-Rad), blocked with 5% skim milk diluted in Tries-buffered saline containing 0.05% Tween-20 (TBST) for 1 h at room temperature, and then incubated with primary antibody overnight at 4°C (anti-SIRT3 antibody, 1:500), incubation with HRP-conjugated secondary antibodies for 1 h at room temperature. Immunoreactive bands and molecular weight were detected using the Odyssey Infrared Imaging System (LI-COR Bioscience, United States). The antibodies used in these experiments are shown in Supplementary Table S1.

### Statistical analyses

All experiments were replicated three or more times; data are presented as the mean ± SD unless otherwise indicated, and all % data were subjected to an arcsine-square-root transformation before statistical analysis. Differences between the two groups were analyzed for statistical significance using two-tailed unpaired Student’s t-tests. Comparisons between more than two groups were analyzed using a one-way ANOVA (Analysis of Variance) test implemented in GraphPad Prism 7 (GraphPad Software, San Diego, CA, United States). *p < 0.05; **p < 0.01, and ***p < 0.001.

## Data Availability

The datasets presented in this study can be found in online repositories. The names of the repository/repositories and accession number(s) can be found in the article/Supplementary Material.
