# Supplementary material for: Metformin promotes in vitro maturation of oocytes from aged mice by attenuating mitochondrial oxidative stress via SIRT3-dependent SOD2ac
Source: Front Cell Dev Biol. 2022 Oct 25;10:1028510. doi: 10.3389/fcell.2022.1028510 (PMC9640937; doi:10.3389/fcell.2022.1028510)
Supplement: Supplementary file 1 [file DataSheet2.PDF]

Table S2 Primer sequences

Gene name	Primers Sequence	Uses
Nutf2	F: GATAACGACAGAACCCAACTAGG<br>R: GGAAGGCTAGATAGCTTCTCCA	qPCR
Bmp15	F: 5'-GTCACCTCTACAATACCGTCCG-3'<br>R: CACCCGGTCCAGGTTAAACA-3'	
Gdf9	F: 5'-GACCTGCCTTACGACTATG-3'<br>R: GAAGAGCGACCTGAGTTG-3'	
Sirt3	F: 5'-TGGGGAGTGGTGCTTTTTATG-3'<br>R: GGGCAATGTAGGGTCGTCAG-3'	
Sod2	F: 5'-AGGTCGGTGTGAACGGATTTG-3'<br>R: TGTAGACCATGTAGTTGAGGTCA-3'	
Uqcrb	F: GGCCGATCTGCTGTTTCAG<br>R: TGATGCCTCATAGTCAGGTCC	
Mrps21	F: AGTGATGGTTCAGGAGGGGAA<br>R: GCTTCTCATAGTAGCGTCGTC	
Gapdh	F: 5'-AAATCAAGGAGTTTGCAGCCGG-3'<br>R: TTCTCTATCACCTGGGGCTCCT-3'	
Sirt3	F: 5'-GCCTCTACAGCAACCTTCA-3'	siRNA
