# Supplementary material for: Metformin promotes in vitro maturation of oocytes from aged mice by attenuating mitochondrial oxidative stress via SIRT3-dependent SOD2ac
Source: Front Cell Dev Biol. 2022 Oct 25;10:1028510. doi: 10.3389/fcell.2022.1028510 (PMC9640937; doi:10.3389/fcell.2022.1028510)
Supplement: Supplementary file 2 [file DataSheet1.PDF]

Table S1. Antibody information

Protein name	Manufacture (catalogue number)	Applications(working dilution)	Uses
Alexa Fluor(anti-rabbit) 488	Cell Signaling(4412S)	1:500	
Anti-a-tubulin-FITC antibody	Sigma(F2168)	1:800	
carboxy-H2DCF diacetate	Beyotime(S0033)	10 mM	
Caspase3	Abcam(ab13847)	1:100	
DAPI	Solarbio(C0060)	1mg/ml	
Fluorescein labeled Lens Culinaris Agglutinin	Vectorlabs(FL-1041-5)	1:200	
In Situ Cell Death Detection Kit	Roche(11684795910)	1:2	Confocal Microscope
LC3	Cell Signaling(4108)	1:300	
Lens culinaris (LCA)-FITC	Vectorlabs(FL-1041-5)	1:200	
MitoProbe™ JC-1 Assay	Invitrogen(M34152)	2 μM	
MitoTracker Red	Invitrogen(M7512)	200 nM	
Mounting Medium with DAPI	Vector(H-1200)	10ml	
SOD2K68ac	Abcam(ab137037)	1:300	
SIRT3	Proteintech(10099-1-AP)	1:500	
TUBLIN	Proteintech(66009-1-Ig)	1:2000	Western Blot
Goat Anti-Rabbit	ZB-2301	1:5000	
